# Car indoor air pollution - analysis of potential sources

**DOI:** 10.1186/1745-6673-6-33

**Published:** 2011-12-16

**Authors:** Daniel Müller, Doris Klingelhöfer, Stefanie Uibel, David A Groneberg

**Affiliations:** 1 Institute of Occupational, Social and Environmental Medicine, Goethe-University, Frankfurt, Germany

## Abstract

The population of industrialized countries such as the United States or of countries from the European Union spends approximately more than one hour each day in vehicles. In this respect, numerous studies have so far addressed outdoor air pollution that arises from traffic. By contrast, only little is known about indoor air quality in vehicles and influences by non-vehicle sources.

Therefore the present article aims to summarize recent studies that address i.e. particulate matter exposure. It can be stated that although there is a large amount of data present for outdoor air pollution, research in the area of indoor air quality in vehicles is still limited. Especially, knowledge on non-vehicular sources is missing. In this respect, an understanding of the effects and interactions of i.e. tobacco smoke under realistic automobile conditions should be achieved in future.

## Introduction

Air quality plays an important role in occupational and environmental medicine and many airborne factor negatively influence human health [1-6]. This review summarizes recent data on car indoor air quality published by research groups all over the world. It also refers to formerly summarized established knowledge concerning air pollution. Air pollution is the emission of toxic elements into the atmosphere by natural or anthropogenic sources. These sources can be further differentiated into either mobile or stationary sources. Anthropogenic air pollution is often summarized as being mainly related to motorized street traffic (especially exhaust gases and tire abrasion). Whereas other sources including the burning of fuels, and larger factory emissions are also very important, public debate usually addresses car emissions.

The World Health Organization (WHO) estimates 2.4 million fatalities due to air pollution every year. Since the breathing of polluted air can have severe health effects such as asthma, COPD or increased cardiovascular risks, most countries have strengthened laws to control the air quality and mainly focus on emissions from automobiles.

**Figure 1:**
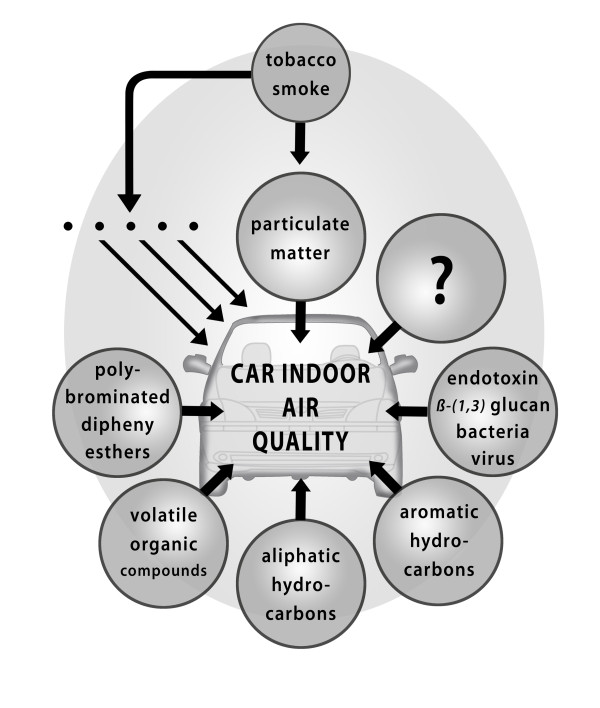
**Factors that can influence indoor air quality in cars negatively**.

In contrast to the amount of research that is currently conducted in the field of health effects, only little is known on specific exposure situations due to external sources which are often present in the indoor environment of a car but not related to the car emissions. The studies addressed a number of vehicular or non-vehicular sources (Figure 1).

## Particulate matter components

One general study assessed the exposure to fine airborne particulate matter (PM_2.5_) in closed vehicles [7]. It was reported that this may be associated with cardiovascular events and mortality in older and cardiac patients. Potential physiologic effects of in-vehicle, roadside, and ambient PM_2.5 _were investigated in young, healthy, non-smoking, male North Carolina Highway Patrol troopers. Nine troopers (age 23 to 30) were monitored on 4 successive days while working a 3 P.M. to midnight shift. Each patrol car was equipped with air-quality monitors. Blood was drawn 14 hours after each shift, and ambulatory monitors recorded the electrocardiogram throughout the shift and until the next morning [7]. Data were analyzed using mixed models. In-vehicle PM_2.5 _(average of 24 μg/m^3^) was associated with decreased lymphocytes (-11% per 10 μg/m^3^) and increased red blood cell indices (1% mean corpuscular volume), neutrophils (6%), C-reactive protein (32%), von Willebrand factor (12%), next-morning heart beat cycle length (6%), next-morning heart rate variability parameters, and ectopic beats throughout the recording (20%) [7]. Controlling for potential confounders had little impact on the effect estimates. The associations of these health endpoints with ambient and roadside PM_2.5 _were smaller and less significant. The observations in these healthy young men suggest that in-vehicle exposure to PM_2.5 _may cause pathophysiologic changes that involve inflammation, coagulation, and cardiac rhythm [7].

A second study by Riedecker et al. assessed if the exposure to fine particulate matter (PM2.5) from traffic affects heart-rate variability, thrombosis, and inflammation [8]. This work was a reanalysis and investigated components potentially contributing to such effects in non-smoking healthy male North Carolina highway patrol troopers. The authors studies nine officers four times during their late shift. PM2.5, its elemental composition, and gaseous copollutants were measured inside patrol cars [8]. Components correlating to PM2.5 were compared by Riedecker et al. to cardiac and blood parameters measured 10 and 15 h, respectively, after each shift. The study demonstrated that components that were associated with health endpoints independently from PM2.5 were von Willebrand Factor [vWF], calcium (increased uric acid and decreased protein C), chromium (increased white blood cell count and interleukin 6), aldehydes (increased vWF, mean cycle length of normal R-R intervals [MCL], and heart-rate variability parameter pNN50), copper (increased blood urea nitrogen and MCL; decreased plasminogen activator inhibitor 1), and sulfur (increased ventricular ectopic beats) [8].

The changes that were observed in this reanalysis were consistent with effects reported earlier for PM2.5 from speed-change traffic (characterized by copper, sulfur, and aldehydes) and from soil (with calcium) [7]. However, the associations of chromium with inflammation markers were not found before for traffic particles. The authors concluded that aldehydes, calcium, copper, sulfur, and chromium or compounds containing these elements seem to directly contribute to the inflammatory and cardiac response to PM2.5 from traffic in the investigated patrol troopers. Interestingly, it was not studied whether other PM2.5 sources that frequently occur in cars such as cigarette smoke have effects at this magnitude.

To understand the dynamics of particulate matter inside train coaches and public cars, an investigation was carried out during 2004-2006 by Nasir and Colbeck [9]. They demonstrate that for air-conditioned rail coaches, during peak journey times, the mean concentrations of PM10, PM2.5 and PM1 were 44 μg/m3, 14 μg/m3 and 12 μg/m3, respectively [9]. They also reported that the levels fell by more than half (21 μg/m3, 6 μg/m3, and 4 μg/m3) for the same size fractions, on the same route, during the off-peak journeys [9]. Also, non-air-conditioned coaches were assessed and it was found that the PM10 concentrations of up to 95 μg/m3 were observed during both peak and off-peak journeys. By contrast, concentrations of PM2.5 and PM1 were 30 μg/m3 and 12 μg/m3 in peak journeys in comparison to 14 μg/m3 and 6 μg/m3 during off-peak journeys [9]. The authors studied particulate air pollution in transport micro-environments over a period of four months and within this period, the concentrations of PM10, PM2.5 and PM1 in car journeys were generally similar during both morning and evening journeys with average values of 21 μg/m3 for PM10, 9 μg/m3 for PM2.5 and 6 μg/m3 for PM1 [9]. However, they also reported that during October the average concentration of PM10 was 31 μg/m3. Interestingly, an analysis of nearby fixed monitoring sites for both PM10 and PM2.5 showed an episode of high particulate pollution over southern England during one week of October. There was no statistically significant difference between particulate matter levels for morning and evening car journeys. A statistically significant correlation was present between morning and evening PM10 (0.45), PM2.5 (0.39) and PM1 (0.46) [9]. The study also showed a statistically significant difference for peak and off-peak levels of PM10, PM2.5 and PM1 in air-conditioned train coaches. On the other hand, in non air-conditioned coaches a significant difference was documented only for PM2.5 and PM1 [9].

Next to PM10 and PM2.5 focussed studies, also ultrafine particles (UFP) have been assessed. In this respect, Liu and colleagues have aimed to quantify exposure to UFP because of second hand smoke (SHS) and to investigate the interaction between pollutants from SHS and vehicular emissions [10]. They measured the number concentration and size distribution of UFP and other air pollutants such as CO, CO2 and PM2.5 inside a moving vehicle under five different ventilation conditions [10]. The vehicle was moved on an interstate freeway with a speed limit of 60 mph and on an urban roadway with a speed limit of 30 mph. It was shown that in a typical 30-min commute on urban roadways, the SHS of one cigarette led to a approximately 10 times increased amount of UFP and 120 times increased amount of PM2.5 in comparison to ambient air [10]. The study indicated that window opening is an effective method for decreasing pollutant exposures on most urban roadways. By contrast some road conditions such as tunnels or crowded freeways with a high proportion of diesel trucks do not allow window opening to be a safe method to decrease UFP levels significantly. In summary, it can be concluded that high ventilation rates may effectively reduce UFPs inside moving vehicles in some road and driving conditions [10].

In parallel, Knibbs et al. assessed on-road and in-vehicle ultrafine (< 100 nm) particle (UFP) concentrations for five different passenger vehicles in an tunnel [11]. They comprised an age range of 18 years. They study encompassed a range of different ventilation settings which were assessed during more than 300 car trips through road tunnel of 4 km in Sydney, Australia [11]. The study quantified the outdoor air flow rates on open roads using tracer gas techniques. It was found that a significant variability in tunnel trip average median in-cabin/on-road (I/O) UFP ratios is present with 0.08 to approximately 1.0. A positive linear relationship was present between outdoor air flow rate and I/O ratio, with the former accounting for a substantial proportion of variation in the latter (R(2) = 0.81). Interestingly, UFP levels recorded in-cabin during tunnel travel were found to be significantly higher than those reported by comparable studies performed on open roadways [11]. Summarizing the data of this study by Knibbs et al. it may be assumed that in-cabin UFP exposures incurred during tunnel travel may contribute significantly to daily exposure under certain conditions. It can also be stated that UFP exposure of automobile occupants appears strongly to be related to the ventilation setting and the vehicle type [11].

## Endotoxin and β-(1, 3)-glucan

A recent study by Wu et al. addressed endotoxin and β-(1,3)-glucan levels in automobiles [12]. This Taiwanese group from the Changhua Christian Hospital, Changhua City, Taiwan postulated that exposure to bacterial endotoxin and fungal β-(1,3)-glucan may also occur in the car indoor environment and can induce major respiratory symptoms. It is known that cars are an exposure source of allergens but it is not specifically known if, and how much exposure there is to fungal β-(1,3)-glucan and endotoxin. Therefore the objective of the project was to assess whether automobiles are a potential source of exposure to these products. Wu et al sampled dust from the passenger seats of 40 cars [12]. A specific Limulus amoebocyte kinetic assays was used to measure endotoxin and β-(1,3)-glucan, respectively. The authors reported that endotoxin and β-(1,3)-glucan were detected in all samples ranging from 19.9-247.0 EU/mg and 1.6-59.8 μg/g, respectively. Significant differences in endotoxin levels between automobiles of smokers and non-smokers were not found, but β-(1,3)-glucan levels were about two-fold higher in the automobiles of non-smokers [12]. It was concluded that endotoxin and β-(1,3)-glucan exposure in automobiles at levels found in this study may be of importance for asthmatics [12].

## Brominated flame retardants

Further substances that may occur in cars are polybrominated diphenyl ethers (PBDEs), hexabromocyclododecanes (HBCDs), and tetrabromobiphenol-A (TBBP-A). A recent project assessed these chemicals in dust from passenger cabins and trunks of 14 UK cars [13]. It was reported that concentrations in cabin dust of HBCDs, TBBP-A, and BDEs 47, 85, 99, 100, 153, 154, 183, 196, 197, 202, 203, 206, 207, 208, and 209 exceeded significantly (p < 0.05) those in trunk dust. The authors concluded that sampling cabin dust appears to provide a more accurate indicator of human exposure via car dust ingestion than trunk dust [13]. Elevated cabin concentrations are consistent with greater in-cabin use of brominated flame retardants (BFRs). In five cars, while no significant differences (p > 0.05) in concentrations of HBCDs and most PBDEs were detected in dust sampled from four different seating areas; concentrations of TBBP-A and of PBDEs 154, 206, 207, 208, and 209 were significantly higher (p < 0.05) in dust sampled in the front seats of the cars [13]. Possible photodebromination of BDE-209 was indicated by significantly higher (p < 0.05) concentrations of BDE-202 in cabin dust. The authors also report that in-vehicle exposure via dust ingestion to PBDEs, HBCDs and TBBP-A exceeded that via inhalation [13]. Comparison with overall exposure via diet, dust ingestion, and inhalation shows while in-vehicle exposure is a minor contributor to overall exposure to BDE-99, ΣHBCDs, and TBBP-A, it is a significant pathway for BDE-209 [13].

## Aromatic hydrocarbons

Next to particulate matter, other noxious compounds including aromatic hydrocarbons, as well as aliphatic hydrocarbons, may play a role in indoor air quality. They diffuse from interior materials in car cabins [14]. In a recent study, seven selected aromatic hydrocarbons were assessed concerning their inhalation toxicokinetics in rats. In brief, amounts of these substances were injected into a closed chamber system containing one rat, and concentration changes in the chamber were examined. Afterwards, toxicokinetics of the substances were analysed on the basis of the concentration-time course using a nonlinear compartment model [14]. Furthermore, the amounts absorbed in humans at actual concentrations in car cabins without ventilation were extrapolated from the results obtained from rats. In specific, the absorbed amounts estimated for a driver during a 2 h drive were as follows per 60 kg of human body weight: 30 μg for toluene, 10 μg for ethylbenzene, 6 μg for o-xylene, 8 μg for m-xylene, 9 μg for p-xylene, 11 μg for styrene and 27 μg for 1,2,4-trimethylbenzene. Concomitantly, in a cabin in which air pollution was marked, the absorbed amount of styrene (654 μg for 2 h in a cabin with an interior maximum concentration of 675 μg/m3) was estimated to be significantly higher than those of other substances [14]. This amount (654 μg) was approximately 1.5 times the tolerable daily intake of styrene (7.7 μg/kg per day) recommended by the World Health Organization [14].

## Volatile organic compounds in car showrooms

Next to exposure inside vehicle, also car dealer showrooms may be places in which occupants may be exposed to emissions from the exhibited vehicles [15]. In order to identify and quantify the main organic compounds present in car dealer showrooms, a total of 19 volatile organic compounds (aromatic compounds, aldehydes and terpenes) were investigated and quantified in showrooms [15]. Also, the levels of the same chemicals were measured in the private houses of the car vendors for comparative purposes. The authors used passive samplers over a consecutive time period of 5 days and reported that the concentrations in the showrooms were on average 12 times higher than the ambient concentration around the showrooms and 10 times higher than the concentrations measured in the private houses. Interestingly benzene concentrations inside the showrooms ranged from 11 to 93.2 μg/m3. It was found that the personal exposure concentrations of the vendors reached time-weighted levels up to 57.3 μg/m3 with minimum values around 10 μg/m3. Overall, this study indicated that work place emissions contribute to a significant proportion of a vendors' overall exposure load [15]. It can be concluded that high concentrations of some volatile organic compounds (VOCs) that were recorded here, point to the importance of occupational safety guidelines for car vendors [15].

Earlier, these authors analysed the presence of selected VOCs including aromatic, aliphatic compounds and low molecular weight carbonyls, and a target set of phthalates in the interior of 23 used private cars during the summer and winter [16]. They reported that VOC concentrations often exceeded levels typically found in residential indoor air, e.g. benzene concentrations reached values of up to 149.1 μg/m3 [16]. Interestingly, overall concentrations were 40% higher in summer, with temperatures inside the cars reaching up to 70 degrees C. The most frequently detected phthalates were di-n-butyl-phthalate and bis-(2-ethylhexyl) phthalate in concentrations ranging from 196 to 3656 ng/m3 [16].

## Microbiological air quality and air conditioning systems

Concerning microbiological air quality and air conditioning systems, Vonberg et al. assessed the impact of air conditioning systems in cars on the number of particles and microorganisms inside vehicles recently [17]. For this purpose, over a time period of 30 months, the quality of air was investigated in three different types of cars which were equipped with an air conditioning system. Different operation modes using fresh air from outside the car as well as circulating air from inside the car were assessed and the total number of microorganisms and mold spores were analysed using impaction in a high flow air sampler [17]. Also particles of 0.5 to 5.0 μm diameter were analysed. In total, 32 occasions of sampling were performed and it was shown that the concentration of microorganisms outside the cars was always higher than it was inside the vehicles [17]. A few minutes after starting the air conditioning system in the cars, it was found that the total number of microorganisms was reduced by 81.7%. Similarly, the number of mold spores decreased by 83.3% [17]. The number of particles was found to be reduced by 87.8%. Interestingly, the authors did not find significant differences between fresh air vs. circulating air conditioning systems or the types of cars. It may be suggested that the use of the car air conditioning system can improve certain parameters of indoor air quality [17].

## Residual tobacco smoke in used cars

Fortmann et al. recently focussed on residual tobacco smoke pollution (TSP) in cars which is caused by frequently smoking cigarettes in a car's microenvironment [18]. They applied surface wipe, air, and dust sampling in used cars sold by non-smokers (n = 40) and smokers (n = 87) and analyzed them for nicotine. Also, primary drivers were interviewed about smoking behaviour [18]. The vehicle interiors were finally inspected to investigate differences in car dustiness and signs of past smoking. Interestingly, smokers reported using air conditioning less (p < 0.05) and driving with windows down more often than non-smokers (p = 0.05) [18]. Also their cars were also dustier (p < 0.01) and exhibited more ash and burn marks than non-smokers' cars (p < 0.001). On further analysis, the number of cigarettes smoked by the primary driver was the strongest predictor of residual TSP indicators (R(2) = .10 - .16, p = 0.001) [18]. Also, this relationship was neither mediated by ash or burn marks nor moderated by efforts to remove residual TSP from the vehicle (i.e., cleaning, ventilation) or attempts to prevent tobacco smoke pollutants from adsorbing while smoking (e.g., holding the cigarette near/outside window) [18]. Two years before, the same group developed and compared methods to measure residual contamination of cars with second-hand smoke in used cars sold by non-smokers (n = 20) and smokers (n = 87) [19]. The sellers were interviewed about smoking behaviour and restrictions, and car interiors were inspected for signs of tobacco use. It was found that the cars of smokers who smoked in their vehicles showed significantly elevated levels of nicotine (p < 0.001) in dust, on surfaces, and in the air compared with non-smoker cars with smoking ban [19]. Also, smoking more cigarettes in the car and overall higher smoking rate of the seller were significantly associated with higher second-hand smoke contamination of the car (p < 0.001) [19]. Interestingly, the use of a cut point for nicotine levels from surface wipe samples correctly identified 82% of smoker cars without smoking bans, 75% of smoker cars with bans, and 100% of non-smoker cars [19]. Thus, it can be concluded that surface nicotine levels provide a relatively inexpensive and accurate method to identify cars and other indoor environments contaminated with residual second-hand smoke [19].

## Conclusion

**Figure 2:**
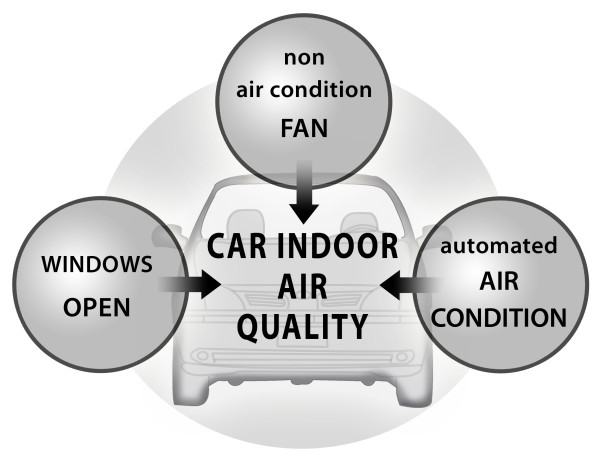
**Factors that may improve indoor air quality in cars when used correctly**.

The quality of the car indoor air may be improved by procedures such as window-opening or the correct use of fans or automated air conditioning systems (Figure 2). In striking contrast to the multitude of studies that address outdoor air pollution, only little is known about indoor air quality in cars. Therefore, modern scientometric tools which are in use for the analysis of other research are not applicable in this area [20-32].

There are numerous approaches present which may bring light to this field of environmental sciences. In specifics, sources and levels of different substances need to be identified and analyzed. Then, further research should be performed about mechanisms, i.e. with the use of modern techniques of biochemistry [33-36], toxicology [37,38] and molecular biology [39-43].

## Competing interests

The authors declare that they have no competing interests.

## Authors' contributions

DM, DK, SU, DAG have made substantial contributions to the conception and design of the review, acquisition of the review data and have been involved in drafting and revising the manuscript. All authors have read and approved the final manuscript.
